# Implementation of superficial radiation therapy (SRT) using SRT‐100 Vision™ for non‐melanoma skin cancer in a Radiation Oncology clinic

**DOI:** 10.1002/acm2.13926

**Published:** 2023-02-17

**Authors:** Yongsook C. Lee, Stephen D. Davis, William Romaguera, Vibha Chaswal, Ranjini Tolakanahalli, Alonso N. Gutierrez, Noah S. Kalman

**Affiliations:** ^1^ Department of Radiation Oncology Miami Cancer Institute Baptist Health South Florida Miami Florida USA; ^2^ Department of Radiation Oncology Herbert Wertheim College of Medicine Florida International University Miami Florida USA

**Keywords:** non‐melanoma skin cancer, SRT‐100 Vision™, superficial radiation therapy (SRT)

## Abstract

**Purpose:**

This article describes our experience in implementation of superficial radiation therapy (SRT) using SRT‐100 Vision™ for non‐melanoma skin cancer.

**Methods:**

Following the American Association of Physicists in Medicine Task Group‐61 protocol, absolute output (absorbed dose to water at surface (cGy/min)) was measured for three energies (50, 70, and 100 kV) and for six applicators (1.5–5.0 cm in diameter). Percent depth dose (PDD) and profiles were also measured. Timer testing and ultrasound testing were performed. A treatment time calculation worksheet was created. Quality assurance (QA) of SRT‐100 Vision was implemented. After treatment workflow for our clinic was developed, end‐to‐end (E2E) testing was performed using a Rando phantom. Considerations for treatment using SRT‐100 Vision were made.

**Results:**

Absolute output (cGy/min) decreases as energy increases and applicator size decreases. Due to scatter from the applicator, PDD at depths ≤5 mm does not follow conventional trends but PDD at depths ≥15 mm increases with increasing applicator size. Profiles for the 5 cm applicator do not have strong dependence on depth except profiles at 5 mm for 50 kV. Timer/end errors are negligible for all three energies. Ultrasound images confirm allowed field of view and depth as well as no image artifacts and spatial integrity. Daily, monthly and annual QA of SRT‐100 Vision implemented in our clinic is listed in a table format. E2E testing results (<1%) demonstrate the functionality and performance of our treatment workflow. Our considerations for SRT treatment include patient, applicator size and energy selections, patient setup, and shields.

**Conclusions:**

This article is expected to serve as guidance for Radiation Oncology and/or Dermatology clinics aspiring to initiate an SRT program in their clinics.

## 1 INTRODUCTION

Non‐melanoma skin cancer (NMSC) is the most frequently diagnosed malignancy in Caucasians and the global incidence of NMSC is continuously rising.^1^, ^2^, ^3^ The most common types of NMSC are basal cell carcinoma (BCC) and squamous cell carcinoma (SCC) with a ratio of approximately 4 to 1 and they are easily curable when detected in early stages.^3^, ^4^ While surgery is still the gold standard for the management of NMSC, various alternative approaches such as radiotherapy, systemic therapy, cryotherapy, and photodynamic therapy are utilized.^1^, ^2^

Radiotherapy plays an integral role in the treatment of NMSC.^5^ Radiotherapy can be performed as a primary treatment when patients are not surgical candidates or have a lesion in a cosmetically sensitive area such as nasal skin, central face, contour of the ear, or eyelids.^2^ Radiotherapy can be also utilized as an adjunct to surgery.^2^, ^5^ Multiple radiotherapy modalities are available to treat NMSC and they include electrons, high‐dose rate brachytherapy, and low‐energy (Grenz and superficial, ≤100 kV) or medium‐energy (orthovoltage, between 100 kV and 300 kV) x‐rays.^6^, ^7^, ^8^, ^9^, ^10^

Owing to its simplicity and cost effectiveness, superficial x‐ray treatment has been used for more than 50 years.^11^ In 2011, Sensus Healthcare (Boca Raton, FL) introduced its superficial radiation therapy (SRT) x‐ray unit named SRT‐100™ to Radiation Oncology and Dermatology clinics. The SRT‐100 is compact, mobile, and remotely controlled. It delivers dose with dose rates comparable to electrons.^11^ Compared with the first generation (SRT‐100 and SRT‐100+™), SRT‐100 Vision™ has more x‐ray energy/treatment modes and notably, it has embedded high‐frequency (20 MHz) ultrasound for imaging.

In this article, we describe our experience in implementation of SRT using the SRT‐100 Vision for treatment of NMSC. The implementation involves (1) commissioning of the unit, (2) quality assurance (QA) of the unit, and (3) treatment workflow with end‐to‐end (E2E) testing. We also present considerations for treatment using the SRT‐100 Vision. To our knowledge, this is the first article describing comprehensive implementation of SRT using the SRT‐100 Vision in a Radiation Oncology clinic.

## 2 METHODS

### 2.1 Commissioning of SRT‐100 Vision

The SRT‐100 Vision has eight energy modes and multiple applicators are available from the manufacturer. Based on our clinical needs, three energies (50, 70, and 100 kV) and six applicators (1.5, 2.0, 2.5, 3.0, 4.0, and 5.0 cm in diameter; source to applicator end distance of 15 cm) were selected for commissioning. The commissioning process is described below. Setups and equipment used for commissioning are listed in Table 1.

**TABLE 1:** Setups and equipment used for commissioning of SRT‐100 Vision.

**Measurement/testing**		**Setup**	**Equipment**
AAPM TG‐61 dosimetry	Half‐value layer	Applicator size: 2.5 cm SDD: 50 cm for Al plates and 100 cm for A11 chamber	Standard Imaging Exradin^®^ A11 parallel plate IC and MAX 4000 electrometer, and aluminum plates
	Relative applicator factors	Applicator size: 1.5–5.0 cm SDD: 25 cm	Standard Imaging Exradin^®^ A26 IC and MAX 4000 electrometer and polyethylene foil
	Absolute output	Applicator size: 5.0 cm SDD: 15 cm	Standard Imaging Exradin^®^ A11 parallel plate IC and MAX 4000 electrometer and polyethylene foil
Beam data collection	Percent depth dose	Applicator size: 1.5–5.0 cm SSD: 15 cm	PTW TN23342A parallel plate IC, Standard Imaging MAX 4000 electrometer, solid water phantoms, and polyethylene foil
	Profiles	Applicator size: 1.5–5.0 cm SSD: 15 cm	EBT3 films, solid water phantoms, and polyethylene foil
Timer testing	Timer linearity	Applicator size: 5.0 cm SDD: 25 cm	Standard Imaging Exradin^®^ A26 IC and MAX 4000 electrometer and polyethylene foil
	Timer/end error		
Ultrasound testing		N/A	Distilled water, ultrasound gel, and solid water phantoms
Creation of treatment time calculation worksheet		N/A	N/A
End‐to‐end testing		Applicator size: 2.0, 2.5, and 3 cm SSD: 15 cm	Rando phantom and nanoDot OSLD

Abbreviations: IC, ion chamber; OSLD, optimally stimulated luminescence dosimeter; SDD, source‐to‐detector distance; SSD, source‐to‐surface distance.

#### 2.1.1 American Association of Physicists in Medicine Task Group (AAPM TG)‐61 dosimetry

**FIGURE 1:**
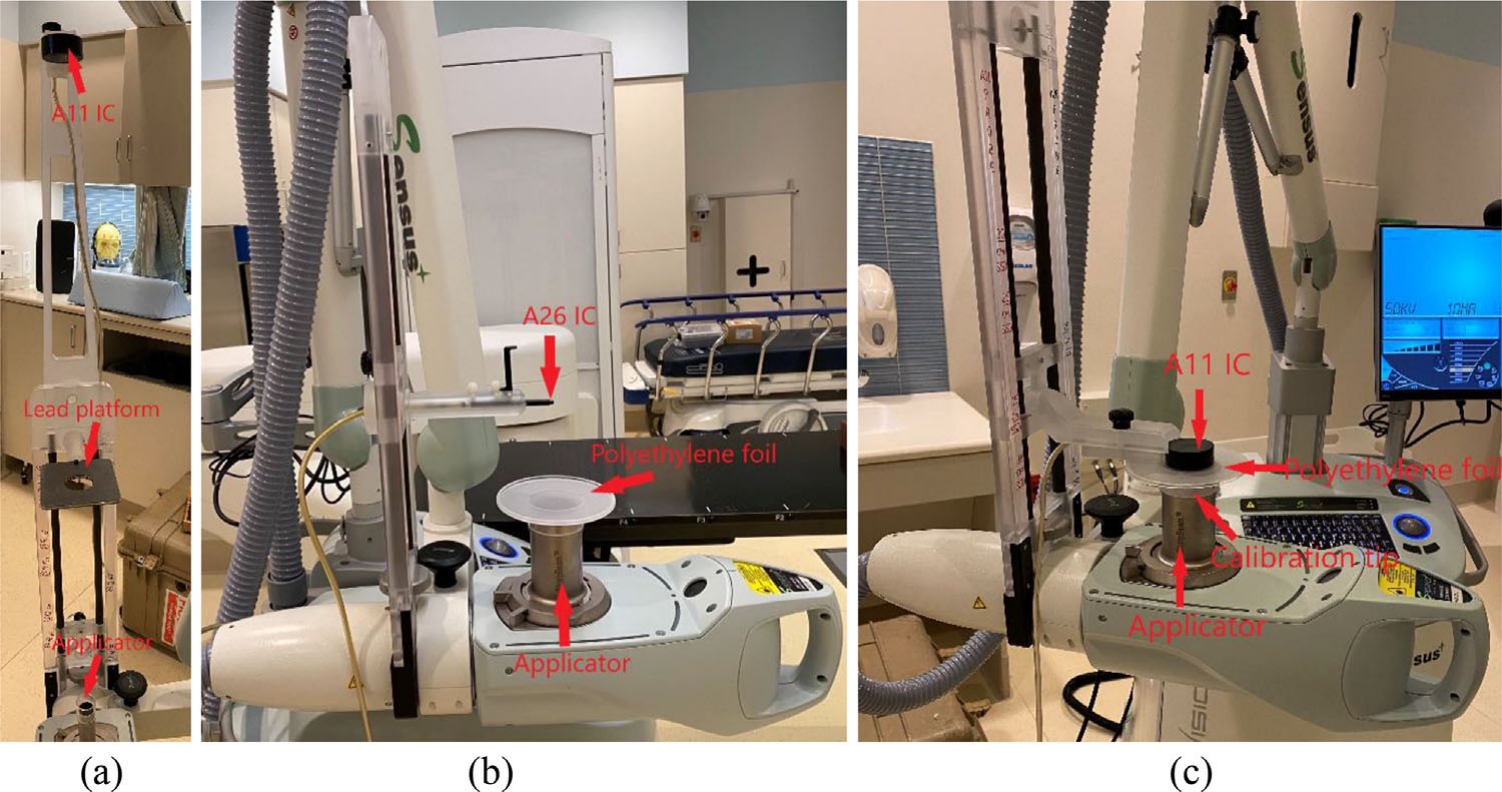
Measurement setups for (a) half‐value layers, (b) relative applicator factors, and (c) absolute output.

A half‐value layer (HVL) was measured and a corresponding air‐kerma calibration factor, $N_{K}$, was determined for each energy. The 2.5 cm applicator, a lead platform with a 4.0 cm aperture and a parallel plate ionization chamber (IC) (Exradin^®^ A11, Standard Imaging, Middleton, WI) were mounted at 15, 50, and 100 cm from the x‐ray source, respectively (Figure 1a). Without and with aluminum (Al) plate(s) on the lead platform, readings (rdgs) (0.5 min per rdg) for 50 kV energy/SRT 50 filter were taken. Then Al thickness to reach [rdg]_Al_/[rdg]_Al=0 mm_ of 0.5 in a semi‐log plot was found. In the same fashion, HVLs for 70 kV/SRT 70 and 100 kV/SRT 100 were measured. $N_{K}\;$for each energy was interpolated from the HVL (mmAl)‐$N_{K}\;\left(\mathrm{Gy}/C\right)$ chart that the Accredited Dosimetry Calibration Laboratory provided for the A11 IC.

Relative applicator factors were measured for each energy. The 5.0 cm applicator and a cylindrical IC (Standard Imaging Exradin^®^ A26) were mounted at 15 and 25 cm from the x‐ray source, respectively (Figure 1b). A circular polyethylene foil (diameter: 10.7 cm) was placed on the applicator (Figure 1b) to eliminate effects of electron contamination. Rdgs (0.5 min per rdg) for 50 kV energy/SRT 50 filter were taken. Measurements were repeated for the rest of the applicators. Relative AFs were determined by taking a ratio of [rdg]_appl_/[rdg]_appl=5 cm_. Relative AFs were also measured for 70 kV/SRT 70 and 100 kV/SRT 100.

Absolute output was measured for each energy following the in‐air method recommended in the AAPM TG‐61 protocol.^7^ The polyethylene foil and a parallel plate IC (Standard Imaging Exradin A11) were placed on the 5 cm applicator (Figure 1c). Temperature and pressure of the room were measured. Rdgs (0.5 min per rdg, $M_{raw}$ in nC) for 50 kV energy/SRT 50 filter were taken with each bias voltage (+300, −300, and +150 V) on. After $P_{TP}$, $P_{ion},$ $P_{pol}$, and corrected rdg, *M*, were calculated using Equations (1), (2), (3), and (4), absorbed dose to water on the phantom surface, $D_{w,z=0}$ in Gy/min, was determined using Equation (5).^7^ The backscatter factor, $B_{w}$ and the ratio for water‐to‐air of the mean mass energy‐absorption coefficients averaged over the photon spectrum, ${\left[{\left(\frac{\bar{\mu_{en}}}{\rho }\right)}_{air}^{w}\right]}_{air}$ were taken from the data in the AAPM TG‐61 protocol.^7^ The chamber stem correction factor, $P_{stem,air}$ was assumed to be unity for calibration. $M\cdot N_{K}$ for the rest of the applicators was determined by multiplying $M\cdot N_{K}$ for the 5.0 cm applicator by relative AFs. Measurements were repeated and $D_{w,z=0}$ in Gy/min, was determined for 70 kV/SRT 70 and 100 kV/SRT 100.

(1) $$P_{TP}=\frac{760\;}{P\;\left(mmHg\right)}\cdot \frac{T\left({}^{\circ }C\right)+273.2}{295.2}$$

(2) $$P_{ion}=\frac{1-{\left(\frac{V_{H}}{V_{L}}\right)}^{2}}{\frac{M_{raw}^{H}}{M_{raw}^{L}}-{\left(\frac{V_{H}}{V_{L}}\right)}^{2}}$$

(3) $$P_{pol}=\left|\frac{M_{raw}^{+}-M_{raw}^{-}}{2M_{raw}}\right|$$

(4) $$M=M_{raw}P_{elec}P_{TP}P_{ion}P_{pol}$$

(5) $$D_{w,z=0}=MN_{K}B_{w}P_{stem,air}{\left[{\left(\frac{\bar{\mu_{en}}}{\rho }\right)}_{air}^{w}\right]}_{air}$$

#### 2.1.2 Beam data collection

**FIGURE 2:**
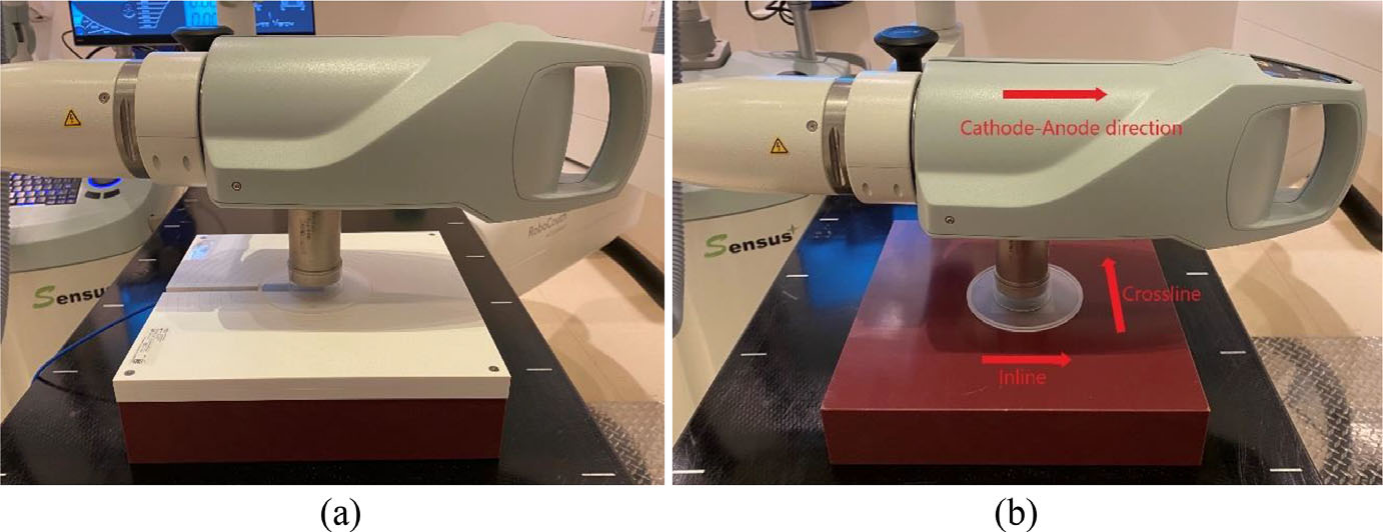
Measurement setups for (a) percent depth dose and (b) profiles.

Percent depth dose (PDD) was measured for each energy and for each applicator. After the polyethylene foil was placed on a parallel plate IC (PTW TN23342A, PTW‐Freiburg, Freiburg, Germany) which was inserted in a RW3 slab phantom (30 cm × 30 cm × 2 cm) (PTW T29672), the 5.0 cm applicator was centered on the IC (Figure 2a). Then Rdgs (0.3 min per rdg) for 50 kV energy/SRT 50 filter were taken. Measurements were repeated at depths of 1, 2, 3, 4, 5, 7, 10, 15, 20, 25, 30, and 40 mm without the foil. Measurements were repeated for the rest of the applicators. Measurements were repeated for 70 kV/SRT 70 and 100 kV/SRT 100. PDD for each applicator was plotted by normalized to a depth of 0 mm.

Profiles were measured for 1.5, 3.0, and 5.0 cm applicators and for each energy. After a piece of an EBT3 film (Ashland LLC., Bridgewater, NJ) and the polyethylene foil were placed on a slab phantom (30 cm × 30 cm × 6 cm) (Virtual Water™, CNMC, Nashville, TN) in order (Figure 2b), the 5.0 cm applicator was centered on the film (Figure 2b). The 50 kV energy/SRT 50 filter beam was delivered to the film for 0.3 min. Measurements were repeated at depths of 1, 3, and 5 mm without the foil. Measurements were repeated for 1.5 and 3.0 cm applicators. Measurements were repeated for 70 kV/SRT 70 and 100 kV/SRT 100. Within 24 h, films were scanned with 300 dots per inch in a scanner (Epson 10000XL, Seiko Epson Corp., Nagano, Japan). After dose and scale calibrations were applied to each film, profiles were read in two perpendicular directions (inline: parallel to cathode‐anode direction; crossline: perpendicular to cathode‐anode direction (Figure 2b) in Image J software (National Institutes of Health, Bethesda, MD).

#### 2.1.3 Timer testing

Timer linearity was measured and a timer/end error was determined for each energy. With the same setup (5.0 cm applicator) and the same IC as for relative AF measurements (Figure 1b), rdgs (0.1 min per rdg) for 50 kV energy/SRT 50 filter were taken. Measurements were repeated for 0.2, 0.3, 0.4, and 0.5 min per rdg. Measurements were repeated for 70 kV/SRT 70 and 100 kV/SRT 100. Charges (pC) as a function of time (min) were plotted and a linear regression line and its *R* ^2^, goodness‐of‐fit measure, were determined for each energy. Also, each linear regression line was extrapolated to zero charge and time (min) for zero charge was found as a timer/end error.

#### 2.1.4 Ultrasound testing

The functionality of ultrasound imaging was tested. Following the vendor's methodology, the inside of the ultrasound probe attached in the unit was filled with distilled water and ultrasound gel was applied in the probe. In high contrast and gray scales, normal skin of a human subject was scanned by adjusting gain (scale: 1–4). On ultrasound images, visibility of skin layers and imaging artifacts were checked. In the high contrast scale, a slab phantom (30 cm × 30 cm × 0.2 cm) was scanned in two directions. The slab phantom was placed on a table for depth scans, whereas the slab phantom was vertically sandwiched between two taller and thicker slab phantoms (30.1 cm × 30.1 cm × 2 cm) for width scans. On ultrasound images, known depth and width (2 mm each) of the slab phantom were measured using the digital measurement tool in the software of the SRT‐100 unit.

#### 2.1.5 Creation of treatment time calculation worksheet

A worksheet for treatment time calculation was created. The worksheet allows our clinic to calculate treatment time (Equation (6)) based on information on prescribed dose (PD) per fraction, treatment depth, energy, and applicator size. Treatment depth, energy and applicator size determine PDD (%). Energy and applicator size determine $D_{w,z=0}$.

(6) $$T\;\left(min\right)=\frac{Prescribed\;dose\;per\;fraction\;\left(cGy\right)}{D_{w,z=0}\;\left(cGy/min\right)\;\times \;PDD\;\left(\%\right)/100}$$

### 2.2 QA of SRT‐100 Vision

After commissioning, daily, monthly and annual QA of the SRT‐100 Vision were implemented (Table 2). Daily QA includes mandatory x‐ray tube warm up, x‐ray output consistency check, safety check, and ultrasound functional check. Monthly QA is similar to daily QA, but the x‐ray output consistency check is performed using a different dosimeter and ultrasound QA such as low contrast, spatial integrity, and image artifact checks is performed. Annual QA includes AAPM TG‐61 dosimetry, beam quality check, and timer testing. Patient data are backed up annually. All the tests and their baselines were generated in our QA platform (RadMachine, RAD formation, New York, NY).

**TABLE 2:** Quality assurance of SRT‐100 Vision

**Daily**
Warm up
X‐ray tube warm up (all available energies)
X‐ray output verification with RAD Check™ (all available energies)
Dosimetry
X‐ray output consistency (all commissioned energies)
Safety
Audio in the unit
Two cameras in the unit
Beam on indicator
Radiation area monitor (if used)
Stop and start buttons on console
Door interlock (beam off)
Ultrasound
Functional check

**Monthly**
Dosimetry
X‐ray output consistency (all commissioned energies)
Safety
Same as for daily
Ultrasound
Low contrast (tumor‐mimicking object vs. surrounding tissue)
Spatial integrity (depth and width)
Image artifact check

**Annual**
Dosimetry
X‐ray output calibration (all commissioned energies)
X‐ray beam quality (all commissioned energies)
Timer testing (all commissioned energies) ▪ Timer linearity ▪ Timer/end error
Patient data backup

### 2.3 Treatment workflow and E2E testing

**FIGURE 3:**
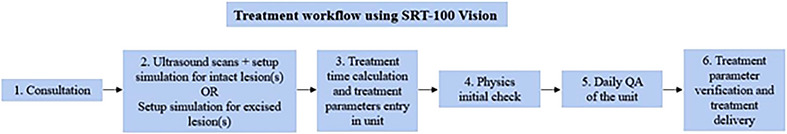
Treatment workflow using the SRT‐100 Vision.

**FIGURE 4:**
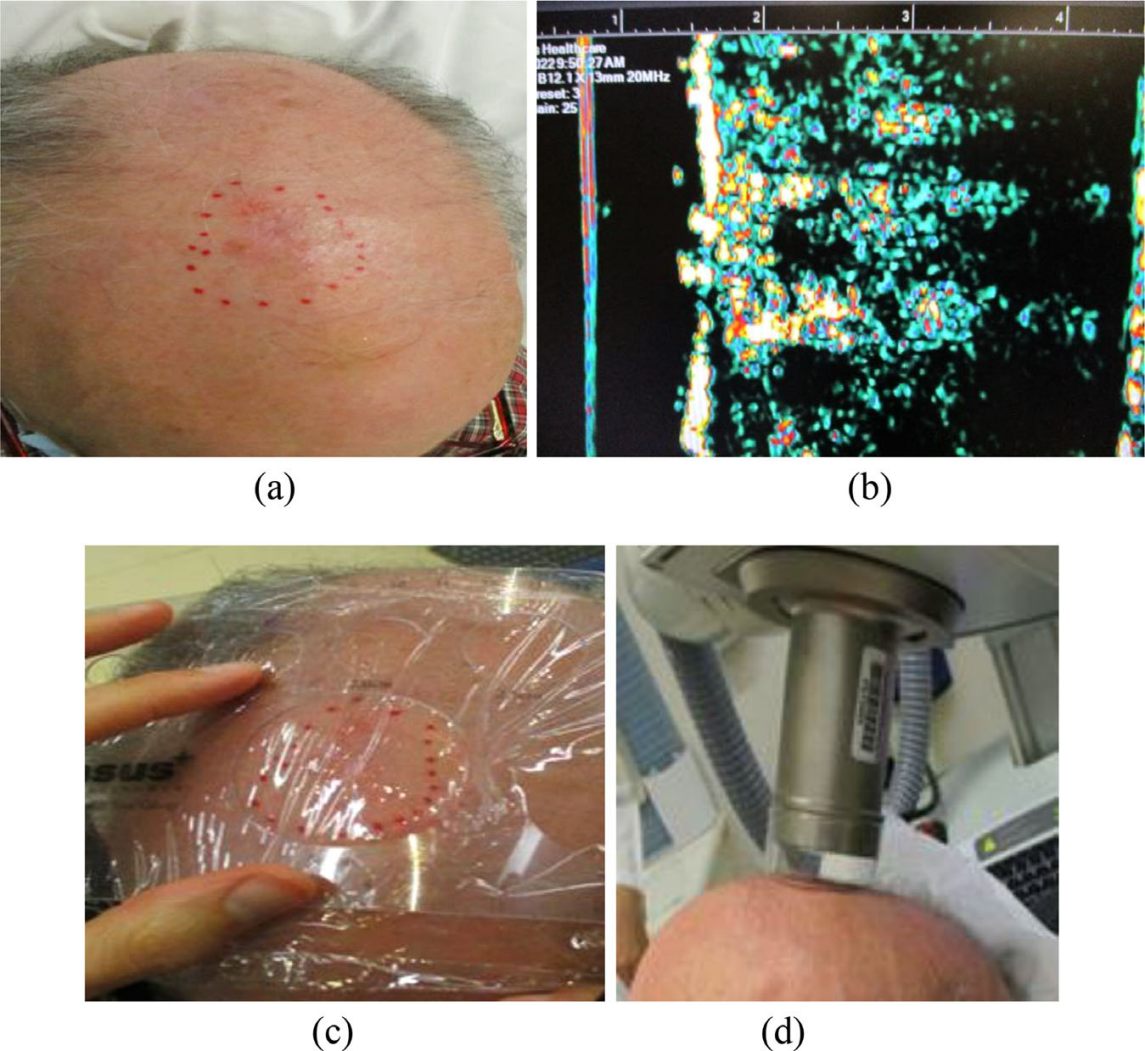
(a) An intact lesion in the scalp, (b) its ultrasound image, (c) applicator size selection, and (d) setup simulation.

Treatment workflow using the SRT‐100 Vision was developed as shown in Figure 3. On the day of simulation, a new patient record is created under the unit's EMR. The radiation oncologist marks the outline of the skin lesion based on clinical and pathologic reports and physical exam (Figure 4a). For intact lesion(s), ultrasound scans are performed by the radiation oncologist to help determine lesion depth and treatment depth (Figure 4b). For excised lesion(s), treatment depth is selected based on clinical and pathologic factors and treatment location. Applicator size is selected based on the longest dimension of the skin lesion as outlined by the radiation oncologist, using the vendor provided applicator‐selector‐tool (Figure 4c). Based on treatment depth and using applicator size specific PDD data, energy (kV) is selected. Then patient setup is simulated using the selected applicator, adequate immobilization devices and internal and/or external shields (Figure 4d). Figure 4 shows a clinical example (SCC in the scalp) of ultrasound scans and setup simulation. After simulation, treatment time is calculated by a dosimetrist and is entered/saved under the patient in the unit. Other treatment parameters such as energy, a total number of fraction and frequency of treatment are also saved under the patient. A physicist independently checks treatment time calculation and treatment parameters entry. On the day of treatment, daily QA is performed by a radiation therapist (Table 2). After the patient is setup, treatment parameters (energy/filter, applicator size, and treatment time) are verified, and treatment is delivered.

**FIGURE 5:**
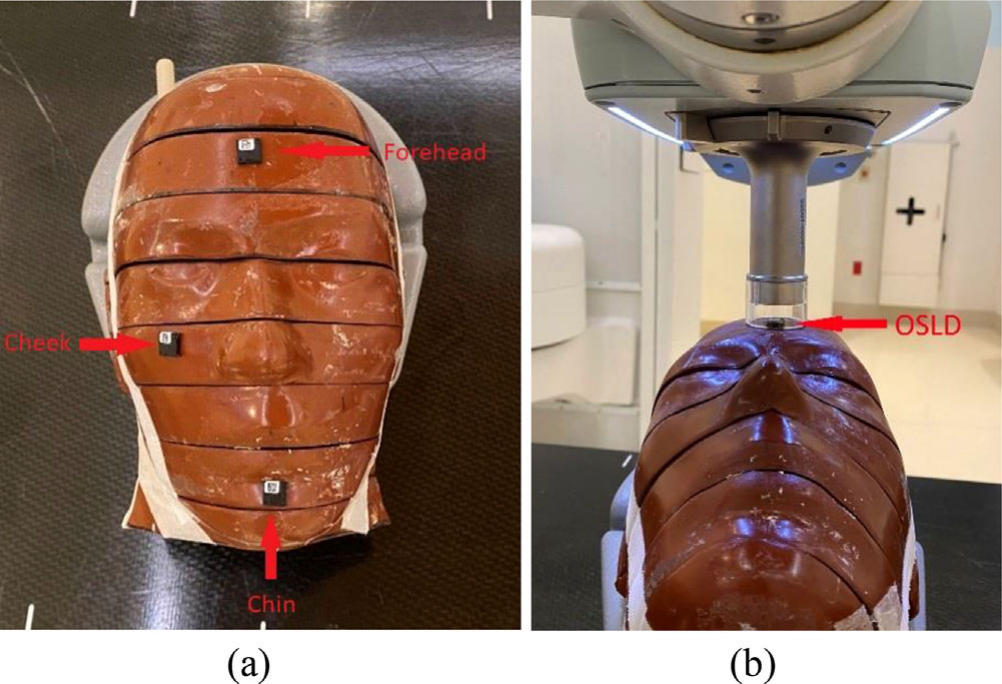
(a) Optimally stimulated luminance dosimeter locations and (b) treatment setup in a Rando phantom for end‐to‐end testing.

Following treatment workflow in Figure 3, E2E testing was performed using a Rando anthropomorphic phantom (Radiology Support Devices, Inc., Long Beach, CA) and nanoDot™ optimally stimulated luminance dosimeters (OSLDs) (Landauer, Glenwood, IL). A patient named Rando phantom was created in the SRT‐100 unit. After setup simulation for three treatment sites (chin, cheek, and forehead) in the phantom, treatment depth, energy, and applicator size were selected (Table 3). Treatment time per fraction (min) for each site was calculated using Equation (6) and was entered/saved in the unit (Table 3). For each site, an OSLD was placed on the Rando phantom (Figure 5a) and the applicator was centered on the OSLD (Figure 5b). After all treatment parameters were verified, treatment was delivered. About 30 min post‐treatment, OSLDs were read out in the Landauer microStar reader device. Expected dose and measured dose on the phantom surface were compared. Before E2E testing, OSLDs were calibrated for each energy.

**TABLE 3:** End‐to‐end testing and its results for three treatment sites of a Rando phantom

**Treatment site**	**Prescribed dose (PD) to treatment depth (cGy)**	**Treatment depth (mm)**	**Energy (kV)**	**Applicator size (cm)**	**Treatment time (min)**	**Expected dose on surface (cGy)**	**Measured dose on surface (cGy)**	**Dose diff. (%) between expected and measured**
Chin	500	1.0	50	2.0	0.72	603	606.24	0.54
Cheek	500	1.5	70	2.5	0.84	580	577.91	0.36
Forehead	500	2.0	100	3.0	0.81	557	557.29	0.05

## 3 RESULTS

### 3.1 Commissioning of SRT‐100 Vision

#### 3.1.1 AAPM TG‐61 dosimetry

Table 4 presents measured HVL (mm Al) and corresponding $N_{K}$ (cGy/nC) for each energy as well as $D_{w,z=0}$ for each energy and for each applicator. As expected, HVL increases as energy increases. $D_{w,z=0}$ decreases as energy increases and applicator size decreases.

**TABLE 4:** Measured half‐value layer, corresponding air‐kerma calibration factor, $N_{K}$ and absorbed dose to water at surface, $D_{w,z=0}$ (cGy/min), for each energy of SRT‐100 Vision

				***D***_***w***, ***z*** = 0_ **(cGy/min)**					
**Energy (kV)**	**HVL (mmAl)**	**Air‐kerma calibration factor,** $N_{K}$ **(cGy/nC)**	**Energy (kV)/App. size (cm)**	**1.5**	**2.0**	**2.5**	**3.0**	**4.0**	**5.0**
**50**	0.461	3.254	**50**	829	839	853	863	871	876
**70**	1.079	3.151	**70**	659	674	691	704	717	727
**100**	1.994	3.093	**100**	633	651	672	688	704	721

#### 3.1.2 Beam data

**FIGURE 6:**
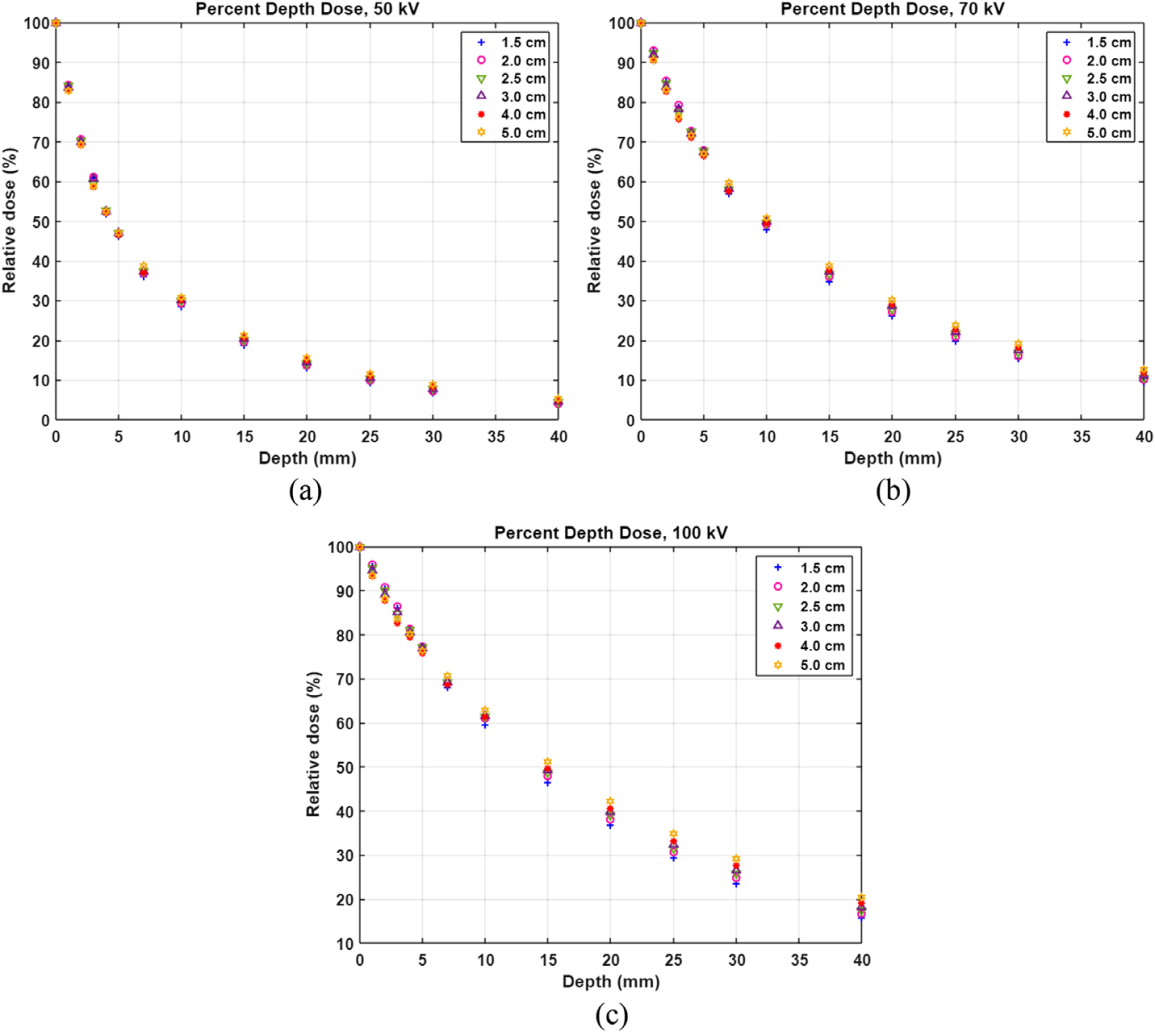
Percent depth dose for (a) 50 kV, (b) 70 kV, and (c) 100 kV.

PDDs for three energies are shown in Figure 6. At depths ≤5 mm, PDD for the 5.0 cm applicator is higher than that for the 4.0 cm applicator but is lower than that for the 3 cm applicator. For applicators ≤3.0 cm, PDD increases and then decreases as applicator size decreases. At depths ≥15 mm, on the other hand, PDD increases with increasing applicator size.

**FIGURE 7:**
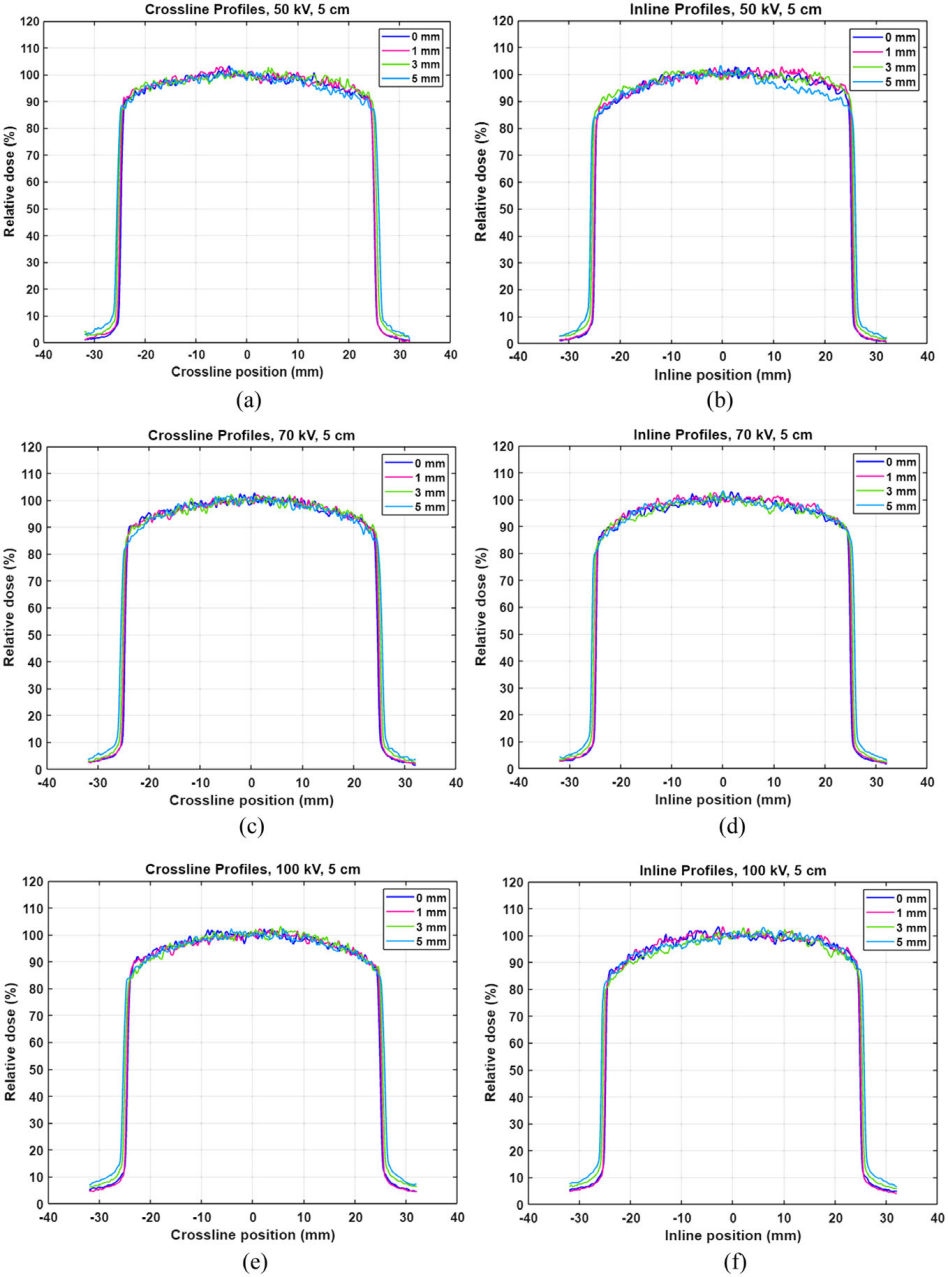
(a,c,e) Crossline and (b,d,f) inline profiles of the 5.0 cm applicator for 50, 70, and 100 kV. Profiles were measured at multiple depths (0, 1, 3, and 5 mm).

Crossline and inline profiles at multiple depths for the 5.0 cm applicator are shown in Figure 7. Relatively greater attenuation is observed in positions between −25 and −10 mm of inline profiles for all three energies. This effect becomes less pronounced as applicator size decreases (profiles for 1.5 and 3.0 cm applicators not shown here). Profiles across the applicator become more uniform as applicator size decreases (data not shown here) and energy decreases. For the 5.0 cm applicator, relative dose of both profiles is ≥90% within ±20 mm (i.e., region excluding a setup margin of 1 cm) (Figure 7). For 1.5 and 3.0 cm applicators, relative dose is ≥95% within ±2.5 and ±10 mm, respectively. Strong dependence of profiles on depth is not observed but more attenuation is observed at a depth of 5 mm for 50 kV (Figure 7a,b). Penumbra (i.e., distance between 20% isodose line (IDL) and 80% IDL) is slightly larger in crossline profiles than in inline profiles for all three energies. Also, penumbra increases as depth increases.

#### 3.1.3 Timer testing

**FIGURE 8:**
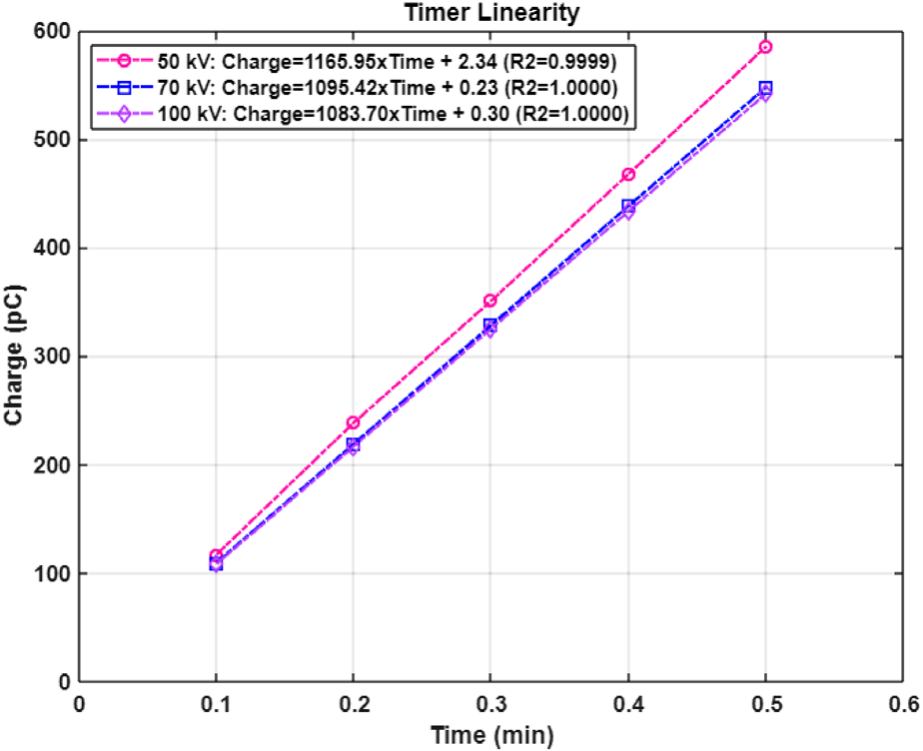
Timer linearity for 50, 70, and 100 kV.

Figure 8 displays linear regression lines along with *R* ^2^ values for all three energies. *R* ^2^ values for all three energies are ≥0.9999, indicating perfect fit. Corresponding timer/end errors are −0.0020, −0.0002, and −0.0003 min for 50, 70, and 100 kV, respectively. The timer/end errors were negligible and were not taken into account in the AAPM TG‐61 dosimetry.

#### 3.1.4 Ultrasound testing

**FIGURE 9:**
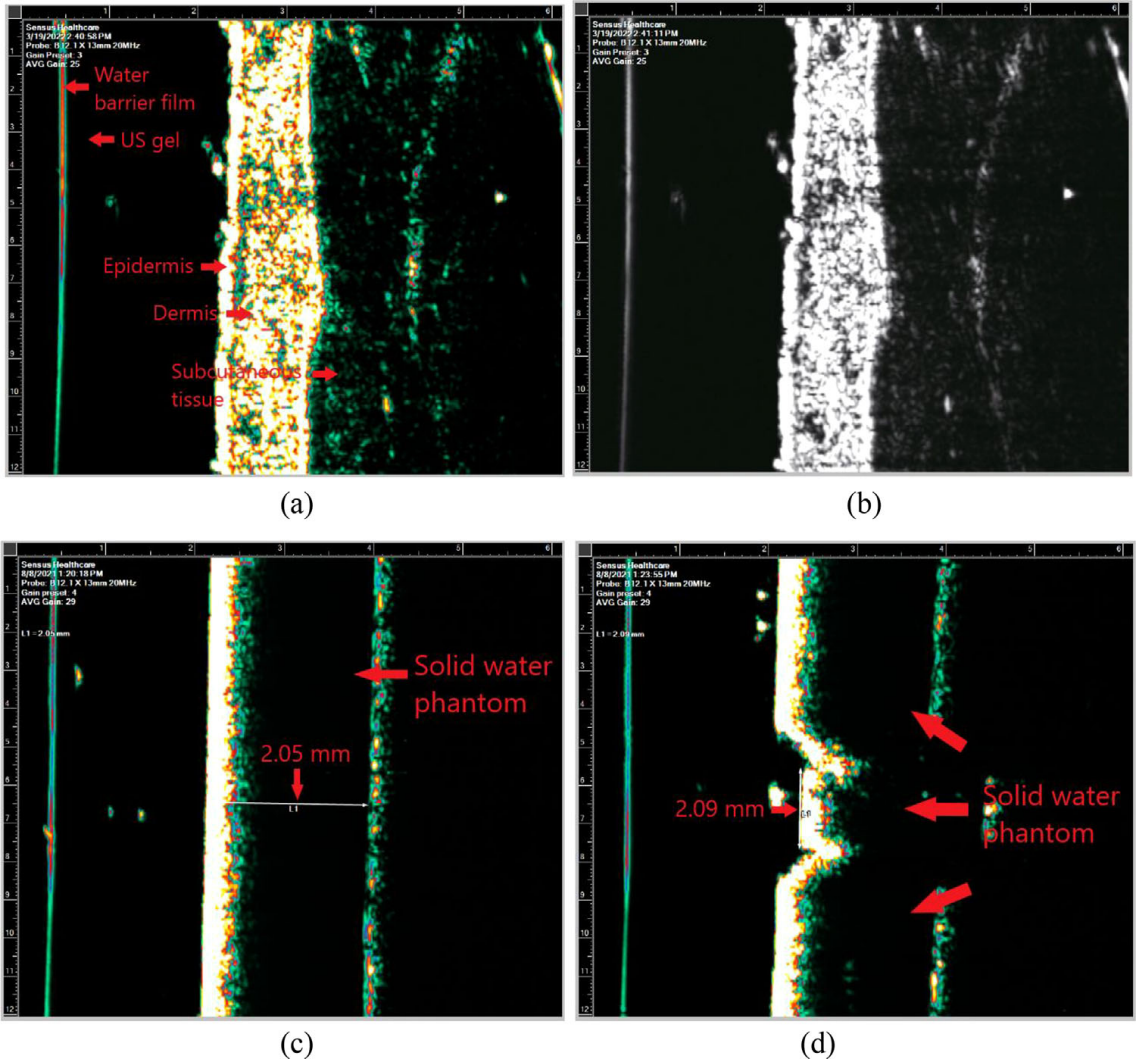
Ultrasound images of normal skin in (a) high contrast and (b) gray scales and ultrasound images of a 2‐mm slab phantom showing (c) depth, and (d) length measurements.

Ultrasound images are shown in Figure 9. The allowed field of view (FOV) is 1.2 cm in length and 4.0–5.0 mm in depth. All the skin layers (epidermis, dermis, and subcutaneous tissue) of a human subject are distinctly visible in high contrast and gray scales (Figure 9a,b). For the best visualization, gain 3 was selected. Measured depth and width of the 2‐mm slab phantom were 2.05 and 2.09 mm, respectively (Figure 9c,d).

### 3.2 E2E testing

Table 3 presents E2E testing results. The differences between expected dose and OSLD measured dose were 0.54%, 0.36%, and 0.05% for 50, 70, and 100 kV, respectively.

### 3.3 Considerations for treatment using SRT‐100 Vision

Considerations for treatment using SRT‐100 Vision in our clinic are summarized in Table 5. Based on our commissioning data, patients who have NMSC with lesion(s) ≤4.0 cm in length and ≤3.0 mm in depth are eligible for treatment. Ultrasound scans are performed for intact lesion(s) to assess radial extent and depth of invasion. Due to FOV limitation, for a lesion >1.2 cm in length, multiple scans are necessary to cover the entire lesion. Applicator size is selected based on the longest dimension of the lesion on skin plus a surrounding setup margin of 0.5–1.0 cm. In addition, profile data are reviewed to ensure adequate target coverage (e.g., at least 90% of PD to the entire lesion at the treatment depth). For elongated large lesion(s), a custom lead shield inside the applicator can be made to protect uninvolved skin. Energy is selected based on the treatment depth. For energy selection, skin dose (<120% of PD) and dose to underlying soft tissues are considered using PDD data for the selected applicator. When lesion(s) around lips are treated, low energy (i.e., 50 kV) is avoided to reduce backscatter from teeth/maxilla/mandible. An internal shield (e.g., lead sheet with wax) inside the mouth can be used to protect the gum regardless of energy selection. During patient setup, the applicator is maneuvered such that x‐ray beam is *en face* and an air gap between skin and applicator is minimized. During treatment, internal shield(s) and/or external shield(s) are used when needed. In addition, patients always wear a lead shield around the neck for thyroid protection.

**TABLE 5:** Considerations for treatment of non‐melanoma skin cancer (NMSC) using SRT‐100 Vision in our clinic

	**Considerations**
Patient selection	▪ NMSC with lesion(s) ≤4.0 cm in length and ≤3.0 mm in depth
Ultrasound scans	▪ Intact lesion(s) ▪ Multiple scans for lesion(s) >1.2 cm in length (FOV: 1.2 cm in length and 4.0–5.0 mm in depth)
Applicator size selection	▪ Longest dimension of a lesion on skin + surrounding setup margin (0.5–1.0 cm) ▪ Target coverage from profile data (e.g., at least 90% of PD to the entire lesion at treatment depth) ▪ For elongated large lesion(s), a custom lead shield inside applicator can be made
Energy selection	▪ Skin dose (<120% of PD) and dose to underlying soft tissues relative to PD at treatment depth ▪ Underlying bone if applicable (higher backscatter with lower energy)
Patient setup	▪ Adequate immobilization ▪ *En face* beam to lesion(s) ▪ Minimized air gap between skin and applicator
Shields	▪ Internal shield(s) (e.g., nasal shield for nose) and external shield(s) (e.g., eye shield) when needed

Abbreviations: FOV, field of view; PD, prescribed dose.

## 4 DISCUSSION

During commissioning, HVL and $D_{w,z=0}$ are measured and entered in the unit. As energy increases, photons are more penetrating and as a result, a thicker HVL is required (Table 4). For each applicator, $D_{w,z=0}$ decreases as energy increases because $N_{K}$ decreases as energy increases (Table 4). For each energy, $D_{w,z=0}$ decreases as applicator size decreases because relative AF and $B_{w}$ decrease as applicator size decreases.

PDD should be measured for each energy of an SRT‐100 Vision unit. As Sheu et al. reported, beam quality would be different from unit to unit and consequently, PDD would be different for each unit.^11^ At depths ≤5 mm, PDD for the 5.0 cm applicator is higher than that for the 4.0 cm applicator for all three energies but PDDs for 2.0, 2.5, and 3.0 cm applicators are higher than PDD for the 5.0 cm applicator and PDD for the 2.0 cm applicator is higher than PDD for the 1.5 cm applicator. This can be explained with scatter. For applicators ≥4.0 cm, phantom scatter reaching IC increases as applicator size increases. For applicators ≤3.0 cm, on the other hand, scatter contribution from the applicator (made of metal) dominates the phantom scatter contribution to the IC relatively, because of the proximity of the applicators to the IC. For the 1.5 cm applicator, due to small field size, phantom scatter contribution decreases further. At deeper depths (≥15 mm), scatter from the applicator reaching the IC is reduced and therefore, the conventional trend (i.e., PDD decreases as applicator size decreases) is observed.

Despite the limitation in film dosimetry, trends of profiles as a function of energy were still observed (Figure 7). Relatively greater attenuation in positions between −25 and −10 mm of inline profiles for the 5.0 cm applicator is attributed to increased attenuation toward the anode, as known also, the heel effect, observed in x‐ray tubes. The heel effect decreases as applicator size decreases because the applicator becomes farther away from the anode with decreasing applicator size. For each applicator, relative dose with respect to central axis dose decreases in the field edge as energy increases. This is attributed to decreased lateral scatter angles with higher energy. For each energy, relative dose decreases in the field edge as applicator size increases (data not shown) because lateral scatter is reduced in the field edge. Due to noisy profiles, it was challenging to observe dependence on depth (Figure 7). The profiles for the 5 cm applicator measured at a depth of 5 mm using 50 kV are noticeably attenuated (Figure 7a,b). This is attributable to low energy (50 kV) and deeper depth (5 mm). Penumbra in inline profiles is slightly smaller than in crossline profiles as the direction of x‐ray production (from cathode to anode) is parallel to inline profiles. Penumbra increases as depth increases (Figure 7) because geometric penumbra ($=\frac{s\cdot (SSD+d-SDD)}{SDD}\;$ where s is source size, SSD is source‐to‐surface distance, d is depth and SDD is source‐to‐diaphragm distance) increases as depth increases.

Using profile data for the selected applicator, target coverage can be confirmed as in Lee et al.’s study.^6^ As an example, a BCC patient is presented with a lesion of 4.0 cm in length and 3.0 mm in depth. If the 5.0 cm applicator is chosen, the edge of the lesion would receive ∼90% of PD (Figure 7). For better target coverage (e.g., >95% of PD), a larger applicator can be selected at the physician's discretion if commissioned.

Daily, monthly and annual QA of the SRT‐100 Vision were established in this article. This is a novel addition since machine QA of an SRT‐100 unit has not been reported in the literature. Like QA of other radiation therapy machines,^12^ dosimetry check and safety check of the SRT‐100 unit are strongly recommended. For annual QA, part of commissioning measurements is repeated for consistency check. Additionally, ultrasound imaging QA should be performed. Since the unit has high frequency (20 MHz) ultrasound, a penetration depth below ultrasound gel is shallow (≤5 mm) (Figure 9). Therefore, a phantom for ultrasound QA should include small tumor‐simulating object(s) (diameter: 2–3 mm) at a shallow depth (≤5 mm) and its contrast and spatial integrity should be checked as in Lee et al.’s study.^13^

E2E testing results (<±1%) confirmed the functionality and performance of our SRT treatment workflow. In our E2E testing, ultrasound scans were skipped as for excised lesion(s) (more frequent in our clinic). Three different treatment sites on the face were selected to use three energies and three applicators. Although it is simple, treatment workflow and E2E testing for SRT using an SRT‐100 unit have not been reported in the literature. During implementation of SRT in any clinic, therefore, own workflow development and E2E testing are recommended before patient treatment.

The considerations listed in Table 5 would help the clinician select the right patient, right applicator size, and right energy. Depending on the location, patient setup can be challenging. An air gap could compromise dosimetry and irradiate uninvolved adjacent area(s). Thus, it is important to minimize an air gap during setup and to use external shields. In this work, our main focus was on treatment of NMSC but other types of cancers in superficial lesion(s) ≤5 mm in depth such as keloids, melanoma, lymphoma, or sarcoma can be treated using SRT.

## 5 CONCLUSION

In this article, we demonstrated successful implementation of SRT using the SRT‐100 Vision from commissioning of the unit, QA program establishment, and workflow management to E2E testing. We also presented our considerations in SRT treatment. With many of these details hitherto unavailable in the literature, this article is expected to serve as guidance for Radiation Oncology and/or Dermatology clinics aspiring to initiate an SRT program using the SRT‐100 Vision in their clinics.

## AUTHOR CONTRIBUTIONS

Yongsook C. Lee designed the work, collected the entire data, and drafted the manuscript. Stephen D. Davis and William Romaguera contributed to the conception of the work and collected data. Ranjini Tolakanahalli and Alonso N. Gutierrez contributed to the conception of the work. Vibha Chaswal and Noah S. Kalman contributed to critical reviews of the manuscript.

## CONFLICT OF INTEREST STATEMENT

The authors claim no conflicts of interest.

## Data Availability

Research data are not shared.
